# Bead-Based Hydrodynamic Simulations of Rigid Magnetic Micropropellers

**DOI:** 10.3389/frobt.2018.00109

**Published:** 2018-09-19

**Authors:** Agnese Codutti, Felix Bachmann, Damien Faivre, Stefan Klumpp

**Affiliations:** ^1^Department Biomaterials, Max Planck Institute of Colloids and Interfaces, Potsdam, Germany; ^2^Department Theory and Bio-Systems, Max Planck Institute of Colloids and Interfaces, Potsdam, Germany; ^3^Biosciences and Biotechnologies Institute (BIAM), CEA Cadarache, Saint Paul Lez Durance, France; ^4^Institute for Nonlinear Dynamics, University of Göttingen, Göttingen, Germany

**Keywords:** micropropeller, randomly shaped, bead approximation, simulation, magnetics

## Abstract

The field of synthetic microswimmers, micro-robots moving in aqueous environments, has evolved significantly in the last years. Micro-robots actuated and steered by external magnetic fields are of particular interest because of the biocompatibility of this energy source and the possibility of remote control, features suited for biomedical applications. While initial work has mostly focused on helical shapes, the design space under consideration has widened considerably with recent works, opening up new possibilities for optimization of propellers to meet specific requirements. Understanding the relation between shape on the one hand and targeted actuation and steerability on the other hand requires an understanding of their propulsion behavior. Here we propose hydrodynamic simulations for the characterization of rigid micropropellers of any shape, actuated by rotating external magnetic fields. The method consists of approximating the propellers by rigid clusters of spheres. We characterize the influence of model parameters on the swimming behavior to identify optimal simulation parameters using helical propellers as a test system. We then explore the behavior of randomly shaped propellers that were recently characterized experimentally. The simulations show that the orientation of the magnetic moment with respect to the propeller's internal coordinate system has a strong impact on the propulsion behavior and has to be known with a precision of ≤ 5° to predict the propeller's velocity-frequency curve. This result emphasizes the importance of the magnetic properties of the micropropellers for the design of desired functionalities for potential biomedical applications, and in particular the importance of their orientation within the propeller's structure.

## 1. Introduction

Controlled propulsion at the micrometer and nanometer-scale is a formidable challenge in robotics. Specifically, propulsion in aqueous environments has tremendous potential for biomedical and environmental applications (Abbott et al., 2009). Over the last years, such microswimmers have been studied extensively (Elgeti et al., 2015; Lauga, 2016) and a number of specific medical applications have been proposed (Peyer et al., 2013; Magdanz and Schmidt, 2014; Sitti et al., 2015; Stanton et al., 2015; Schwarz et al., 2017). In many cases these robots are biohybrids, based on biological swimmers, i.e., bacteria or other living cells that provide the motorization of the propulsion. In a particularly striking example, biohybrid microswimmers based on magnetotactic bacteria have been successfully employed *in vivo* for targeting and penetrating solid tumors (Ouajdi et al., 2016) or to kill biofilms (Stanton et al., 2017). At the moment, biological microswimmers are superior to synthetic ones in many respects, but over the last decade the synthesis and characterization of synthetic microswimmers has made much progress and eventually, which kind of microswimmer is best suited as a robot will depend on the type of application that is envisioned. Synthetic swimmers may for example be preferable in environmental conditions that are toxic to biological swimmers or if the unavoidable biological variability of properties needs to be suppressed.

Currently, one of the most promising non-biological propulsion mechanism is based on actuation of a rigid magnetic object (called a magnetic micropropeller) with a rotating magnetic field, as this mechanism is non-toxic (and could thus be used in biomedical settings) and works remotely, without the need for direct contact with the robot. Rotation of a magnetic field results in propulsion if the micropropeller has a rotation-translation coupling, which usually requires chirality of the structure. Therefore, the first magnetic micropropellers were magnetic helices, inspired by the propulsion of bacteria due to the rotation of their flagella (Ghosh and Fischer, 2009; Zhang et al., 2009; Man and Lauga, 2013). Only in the last years, other propeller designs have been explored. As the range of envisioned tasks is expanding, diverse designs may be required. Non-helical shapes such as a three-beads cluster (Cheang et al., 2014) or v-shaped propellers (Sachs et al., 2017; Tottori and Nelson, 2018) have been proposed. Moreover, studies of random aggregates of magnetic nanoparticles, which are easy and inexpensive to produce, have shown that a wide range of shapes can be propelled and that many of them swim just as well as the carefully designed propellers (Vach et al., 2013, 2015; Bachmann et al., 2018). Since shapes of magnetic microswimmers can also realized by 3D-printing, there is much potential for customization and fine-tuning of diverse shapes.

With the expansion of the design space of possible magnetic propellers, the computational prediction of the behavior of customized shapes becomes highly desirable, which however remains a challenging task from the theoretical point of view despite decades of study of low-Reynolds-number hydrodynamics (Elgeti et al., 2015; Lauga, 2016). Analytical solutions are only possible for simple shapes (Ghosh et al., 2012, 2013; Keaveny et al., 2013; Man and Lauga, 2013; Morozov and Leshansky, 2014; Xu et al., 2016; Morozov et al., 2017), or for slender clusters (Morozov and Leshansky, 2014; Morozov et al., 2017; Mirzae et al., 2018). When analytical solutions are not possible, hydrodynamic simulations can be used to describe the propeller behavior. In general, to describe the dynamics of a micropropeller, it is necessary to know its mobility matrix, which is defined by the geometry of the propeller. This matrix describes the linear relation between the applied forces and torques and the resulting translational and angular velocities. To obtain the mobility matrix for complex geometries, a coarse graining method is required. Here we use an approach that approximates the micropropeller geometry with a cluster of nanometer-sized beads. Such discretization has been used extensively to study propulsion by bacterial flagella and flexible filaments (Manghi et al., 2006; Reichert, 2006), as well as other rigid microswimmers (Carrasco and de la Torre, 1999; Filippov, 2000; Reichert, 2006; Morozov et al., 2017; Mirzae et al., 2018). Here we apply this method to rigid magnetic object using a projection method Reichert (2006).

We systematically study the influence of model parameters (bead size, distance between beads down to touching beads, magnetic moment orientation) on the swimming behavior to identify optimal simulation parameters. For the validation and parametrization of our simulations, we focus on the helical geometry, as this is the best described behavior so far. We observe velocity reversals upon a change of the rotation frequency of the magnetic field, a feature that was recently predicted theoretically for helices (Morozov et al., 2017), but was so far not reported experimentally. We then describe a method to obtain bead representations from three-dimensional reconstructions of experimental propeller shapes and apply this method to a propeller shape that was recently characterized experimentally within a study of randomly shaped propellers (Bachmann et al., 2018). We show that the orientation of the magnetic moment has a strong influence on the dynamics of the propeller. As a consequence, knowing the geometry of the rigid propeller is not enough to fully predict its swimming behavior, but the magnetic moment needs to be characterized as well. As this is often difficult to achieve experimentally, simulations can alternatively be used to determine the magnetic moment by matching the simulated dynamics to the observed one.

## 2. Computational methods

### 2.1. Theoretical framework

To simulate the propulsion of micropropellers of arbitrary shape, Stokesian dynamics (Brady and Bossis, 1988) with a projection method for rigid clusters of particles (Reichert, 2006) are used. In this approach, propellers are approximated as rigid clusters of hard spheres. This method combines versatility (since any shape can be discretized in this way) with simplicity (using the hydrodynamic description for spheres via the method of reflections). In the low Reynolds number regime of hydrodynamics that applies to micro- or nanoscopic objects such as the micropropellers, the velocity and angular velocity of a particle are linearly dependent on the forces and torques experienced by that particle. This linear dependence is characterized by a mobility matrix that fully accounts for the hydrodynamic coupling between different particles or different degrees of freedom (such as rotation and translation) of one particle (Dhont, 1996).

For a rigid object, the motion is described by the translation of its center of mass and the rotation of the body with respect to its center of mass. Thus, the equations of motion are:

(1) $$\begin{array}{l} \left(\begin{array}{c} {\text{v}}_{\text{CM}}\\ \omega_{\text{CM}} \end{array}\right)=\left(\begin{array}{cc} {\text{M}}^{\text{tt}} & {\text{M}}^{\text{tr}}\\ {\text{M}}^{\text{rt}} & {\text{M}}^{\text{rr}} \end{array}\right)\left(\begin{array}{c} {\text{F}}_{\text{CM}}\\ {\text{T}}_{\text{CM}} \end{array}\right). \end{array}$$

Here, **v**_CM_ is the translational velocity of the center of mass, ω_CM_ the angular velocity, connected to the propeller frequency *f*_CM_ via ω_CM_ = 2π*f*_CM_, and **F**_CM_ and **T**_CM_ are the external force and torque, respectively, applied on the center of mass. The **M**^*ij*^ are the 3 × 3 submatrices of the 6 × 6 mobility matrix, the superscripts indicate whether they describe the translational motion ('tt'), rotation ('rr') or the coupling of translation and rotation ('rt' and 'tr'). The mobility matrix is symmetric, thus **M**^rt^ = (**M**^tr^)*^T^*.

In the case of magnetic micropropellers that are rotated by a (spatially homogeneous) magnetic field, the force is usually zero and the external torque is given by **T**_CM_ = **m** × **B**, with the magnetic moment **m** of the propeller and the magnetic field **B**, which is taken to rotate counterclockwise in the *yz*-plane in the lab frame of reference, **B** = (0, *B*cos(2π*f*_B_*t*), *B*sin(2π*f*_B_*t*)). *f*_B_ is the frequency of the field and *B* its absolute value. For the rotation in the *yz*-plane, a net movement of the cluster along the *x*-axis is expected. Since only a torque and no external forces act on the propeller, the geometry of the propeller must have a non-zero rotation-translation coupling, *M*^tr^ ≠ 0 in order to lead to a non-zero translational velocity **v**_CM_ ≠ 0.

The mobility matrix for the center of mass of the propeller can be determined from the mobility matrix of the individual beads describing the discretized shape of the propeller by applying a projection method (Hinsen, 1995; Reichert, 2006), as explained in the following. The 6*N* × 6*N* mobility matrix for *N* beads appears in the equations of motion for the beads,

(2) $$\begin{array}{l} \left(\begin{array}{c} \text{v}\\ \omega \end{array}\right)=\left(\begin{array}{cc} \mu^{\text{tt}} & \mu^{\text{tr}}\\ \mu^{\text{rt}} & \mu^{\text{rr}} \end{array}\right)\left(\begin{array}{c} \text{F}\\ \text{T} \end{array}\right) \end{array}$$

where **v** and **ω** are the 3*N*-dimensional translational and angular velocity vectors of the beads. **F**, **T** are the corresponding (likewise 3*N*-dimensional) vectors of the forces and torques, respectively, that are applied on the individual beads. The **μ**^*ij*^ are 3*N* × 3*N* mobility matrices (with superscripts “t” and “r” for translational and rotational degrees of freedom as described above). The elements of the **μ** matrices can be calculated through the Rotne-Prager approximation (Rotne and Prager, 1969; Dhont, 1996; Reichert, 2006), which leads to equations that are exact up to order ${\left(a/r_{ij}\right)}^{3}$ (Reichert, 2006) (with the bead radius *a* and the distance *r*_*ij*_ between two beads). In the present paper, rotational-translational couplings μ^tr^, μ^rt^ are neglected, which are known to result in artifacts for elastic structures (Reichert, 2006), as well as the off-diagonal terms $\mu_{i\neq j}^{\text{rr}}$ (Reichert, 2006) (see section 2.3). Then, the non-zero entries of the mobility matrix are

(3) $$\begin{array}{l} \mu_{ii}^{\text{tt}}=\mu^{\text{t}}1 \end{array}$$

(4) $$\begin{array}{l} \mu_{ii}^{\text{rr}}=\mu^{\text{r}}1 \end{array}$$

(5) $$\begin{array}{l} \mu_{i\neq j}^{\text{tt}}=\mu^{\text{t}}\left[\frac{3}{4}\frac{a}{r_{ij}}\left(1+{\hat{\text{r}}}_{ij}{\hat{\text{r}}}_{ij}\right)+\frac{1}{2}{\left(\frac{a}{r_{ij}}\right)}^{3}\left(1-3{\hat{\text{r}}}_{ij}{\hat{\text{r}}}_{ij}\right)\right], \end{array}$$

where *i, j* = 1, …, *N* is the index of the bead, **1** is the 3 × 3 identity matrix, μ^t^ = (6π*ηa*)^−1^, μ^r^ = (8π η*a*^^3^)−1^ are the mobility coefficients of a sphere, η is the kinematic water viscosity, *a* is the radius of the bead, *r*_*ij*_ is the distance between the *i*-th and *j*-th bead, and ${\hat{\text{r}}}_{ij}=\frac{{\text{r}}_{i}-{\text{r}}_{j}}{r_{ij}}$ is the unit vector connecting the *i*-th and *j*-th bead. ${\hat{\text{r}}}_{ij}{\hat{\text{r}}}_{ij}$ is a 3 × 3 matrix, in which the elements ${\left[{\hat{\text{r}}}_{ij}{\hat{\text{r}}}_{ij}\right]}_{lk}={\hat{\text{r}}}_{ij}\left(l\right){\hat{\text{r}}}_{ij}\left(k\right)$, where ${\hat{\text{r}}}_{ij}\left(1\right)=x_{ij}$, ${\hat{\text{r}}}_{ij}\left(2\right)=y_{ij}$ and ${\hat{\text{r}}}_{ij}\left(3\right)=z_{ij}$.

The mobility matrix **M** for the center of mass is obtained from the mobility matrix ***μ*** for the individual beads by projecting onto the center of mass (Hinsen, 1995; Reichert, 2006)

(6) $$\begin{array}{l} \text{M}={\left(C^{\text{T}}\cdot \mu \cdot C\right)}^{-1}, \end{array}$$

with the 6 × 6*N* projection matrix

(7) $$\begin{array}{l} C=\left(\begin{array}{cc} 1 & \left({\text{r}}_{\text{c}}-{\text{r}}_{1}\right)\times \\ \vdots & \vdots \\ 1 & \left({\text{r}}_{\text{c}}-{\text{r}}_{N}\right)\times \\ 0 & 1\\ \vdots & \vdots \\ 0 & 1 \end{array}\right), \end{array}$$

Here $r_{\text{c}}=\frac{\underset{i}{\sum }{\text{r}}_{i}}{N}$ is the position of the center of mass and × indicates the vector product. For given external force and torque on the center of mass, the translational velocity **v**_CM_ and angular velocity ***ω***_CM_ of the propeller can then be calculated from equation (1). The position of the center of mass **x**_CM_ is then obtained by integration of

(8) $$\begin{array}{l} \frac{\text{d}{\text{x}}_{\text{CM}}}{\text{d}t}={\text{v}}_{\text{CM}}. \end{array}$$

The orientation of the object is described by a triad of orthogonal unit vectors $\hat{\alpha },\hat{\beta },\hat{\gamma }$ rigidly attached to the body, which rotates due to the angular velocity of the propeller, according to

(9) $$\begin{array}{l} \frac{\text{d}\hat{\alpha }}{\text{d}t}=\omega_{\text{CM}}\times \hat{\alpha } \end{array}$$

and likewise for ***β***. ***γ*** is obtained from the other two vectors through orthogonality.

### 2.2. Implementation of the dynamics

The simulation was implemented in Fortran 90 (Press et al., 1992), based on the following algorithm: the mobility matrix of the center of mass is calculated once in the body system at the beginning of the simulation (Reichert, 2006) and is fixed in the body system. At each time-step, the instantaneous body system is considered, where the mobility matrix is known and the magnetic moment is fixed. The torque is calculated in this system, too. From the torque, the velocity and frequency in the instantaneous body system can be derived via Equation (1). The displacement of position and of the unitary vectors are computed in this body system from integration of Equations 8 and 9 using the second-order Runge-Kutta scheme (Press et al., 1992), and then transformed into the lab system, in which the new position of the center of mass and the new orientation of the triad are obtained as the present-time value plus the corresponding displacement. For Visual Molecular Dynamics (VMD) videos, the position of the beads in the lab system is calculated at each time-step from their fixed position (*a*_*i*_, *b*_*i*_, *c*_*i*_) in the body system defined by $\hat{\alpha },\hat{\beta },\hat{\gamma }$ as ${\text{x}}_{i}={\text{x}}_{CM}+a_{i}\hat{\alpha }+b_{i}\hat{\beta }+c_{i}\hat{\gamma }$. Alternatively, all calculations can be done in the lab system, but then the mobility matrix needs to be re-calculated at each time-step, because of its dependence on the orientation in the lab frame. Thus, this approach requires much longer computation times, but the results of the two algorithms are equivalent. In both approaches, we included a check of the orthogonality of the triad $\hat{\alpha },\hat{\beta },\hat{\gamma }$ over time, because Euler integration of the unit vector ${\hat{\alpha }}_{\text{new}}={\hat{\alpha }}_{\text{old}}+\omega_{\text{CM}}\times {\hat{\alpha }}_{\text{old}}\text{d}t$ does not preserve orthogonality: if ${\hat{\alpha }}_{\text{old}}\cdot {\hat{\beta }}_{\text{old}}=0$, then after integration ${\hat{\alpha }}_{\text{new}}\cdot {\hat{\beta }}_{\text{new}}=-\text{d}t^{2}\left({\hat{\beta }}_{\text{old}}\cdot \omega_{\text{CM}}\right)\left({\hat{\alpha }}_{\text{old}}\cdot \omega_{\text{CM}}\right)$. This problem is kept under control by the second order Runge-Kutta scheme for integration as well as a small time-step d*t* ≃ 10^−5^*s*. Simulations were stopped if the error accumulated to $\hat{\alpha }\cdot \hat{\beta }>10^{-2}$.

### 2.3. Discussion of approximations

The model implies a series of approximations: (i) The continuous surface is approximated with beads; (ii) The Rotne-Prager equations are an approximation themselves, valid for spheres at sufficiently large distances, but used here also for rather small distances (spheres touching each other); (iii) Some terms in the equations are neglected, in particular the rotational-translational terms and the off-diagonal rotational-rotational terms; (iv) Rigid bonds correction were not used. We will go through these points in the following.

- The approximation with beads of a continuous surface was previously used in the literature (Carrasco and de la Torre, 1999; Filippov, 2000; Reichert, 2006; Morozov et al., 2017). Artifacts can arise due to gaps between the beads. Since the aim is a qualitative description of the behavior of the propeller and not the exact flow of the fluid around the object, this problem is of minor relevance.
- The Rotne-Prager approximation is also common in the literature. It is good for spheres that are sufficiently distant from each other (Dhont, 1996; Reichert, 2006) due to lubrication problems that could arise when the gap between them is too small. For a rigid cluster though, lubrication problems are not present, since the spheres are fixed with respect to each other. There are order *O*(1) effects (Dhont, 1996) that are considered negligible in the simulation, since tuning the number of spheres (see section 3.2) caused no significant difference for distant or touching beads. Moreover, these terms would influence only the near-surface flow, but not the overall behavior of the object that we want to represent with an effective motion. Thus, these expressions for the entries of the mobility matrix will be used here also for spheres touching each other.
- The rotational-translational terms and the off-diagonal rotational-rotational terms are neglected following the approach of Reichert (2006), where this was done consistently for flexible chains.
- Rigid bonds corrections have not been considered in our approach. This was done in ref. (Reichert, 2006) through the HYDROLIB library (Hinsen, 1995). Here, instead we used self-written code without the use of that library. Compared to the other approximations we made (such as the discretization), these corrections are, however, of minor importance.

Overall these approximation can lead to quantitative changes in the velocity-frequency curves, but with the qualitative behavior unchanged (see the Supplementary Information).

### 2.4. Bead representation of propeller shapes

To run the simulation, the shape of the propeller must be approximated by beads. How this discretization is done depends on the shape of the propeller itself. For helices, the geometry is defined by the pitch and the radius of the helix and the number of turns. The distance between the beads has been varied, but was eventually chosen to be as close as possible to beads touching each other, in this way selecting the number of beads for a certain radius. The beads are then equally distributed along the helix. The size of the bead influences the number of beads, and also the helix thickness. In all helix simulations we used a helix radius *r* = 2.5 μm, a pitch *p* = 4*r*, and a number of turns *n* = 4. The radius of the beads, *a*, and the number of beads per turn were varied. The basic geometry presents *a* = 0.1 μm with touching beads, if not stated differently.

To model the random-shaped propellers of Bachmann et al. (2018) as clusters of beads, an initial bead configuration must be determined. To do so, the shape of the rotating propeller is obtained with a three dimensional tomographic reconstruction from 2D microscope images by Bachmann et al. (2018). The best approximation would be to assign one bead to each voxel (3D pixel), but the number of beads can be reduced to run faster simulations. Through appropriate binning and thresholding, the propellers can be approximated and coarse-grained, reducing the total number of pixels in the 3D reconstruction. The position of each pixel obtained after binning and thresholding has been taken as the center of a bead, and the radius as half of the pixel size (see section 4). In this way the procedure of reconstruction has been automatized, providing a reproducible way to approximate any shape with beads. Different numbers of beads can be chosen to see the effects of discretization: from a number of beads around 50, for which the features of the propeller can still be seen and not losing much detail, to a number of beads around 500 and then one bead for each voxel (order of thousands). The beads are not only positioned on the surface, but also inside to avoid empty spaces that could lead to unwanted interactions. Higher numbers of beads slows down the generation of output for videos, since the position of every bead must be calculated at each time-step, but not the integration of the equations of motion, on which the number of beads has no influence. The number of beads only affects the initial calculation of the mobility matrix.

Finally, random-shaped propellers can be generated computationally rather than reconstructed from experimental random shapes: a first bead is generated, and then the following beads are randomly attached to the previous once, considering volume exclusion. An example is given in the Supplementary Information.

### 2.5. The determination of the magnetic moment

The magnetic moment can be directed in any orientation with respect to the propeller body. To reproduce the experimental data, the orientation in the real propeller must be known, which is typically not the case for random-shaped propellers, which cannot easily be isolated from the bulk sample for experimental determination of the magnetic moment. Even if isolation is successful, determining the exact orientation of the magnetic moment inside the propeller geometry with the required precision would be difficult. Therefore, the direction of the magnetic moment is determined by analyzing videos that are taken at low frequencies of the magnetic field, when the propeller is aligned in the plane of the field rotation, using a cylindrical approximation. We use the magnetic moment norm and direction that were determined by Bachmann et al. (2018) with the cylindrical approximation according to the method previously reported (Ortega and de la Torre, 2003; Ghosh et al., 2013). With this method, an elongated propeller is approximated by a cylinder, and knowing the propeller parameters (such as how the propeller is oriented in the magnetic field and its characteristic frequencies), the magnetic moment is determined in the hydrodynamic center reference system (Ghosh et al., 2013; Morozov and Leshansky, 2014) and is then rotated to the lab reference system of the simulation. This method to determine the magnetic moment works well when the propeller has an elongated shape. Still, the orientation obtained is not the exact one, but rather an equivalent orientation if the propeller would be a cylinder. This introduces further error in the final result of the simulation compared to experimental results.

We note that here we consider only propellers that possess a permanent magnetic moment, a good description for many experimentally-realized rigid magnetic swimmers (Vach et al., 2013, 2015; Vach, 2015). Propellers based on paramagnetic or superparamagnetic materials have also been studied and lead to different interesting behaviors. Examples include paramagnetic spheres moving on surfaces (Martinez-Pedrero et al., 2015; Helgesen, 2018) or superparamagnetic beads used as biosensors (Janssen et al., 2009).

### 2.6. Initial configurations and branching

For some propellers and for certain frequencies, we observe two stable solutions of the equations of motion at the same field strength and rotation frequency (branching). These correspond to two different orientations of the axis of rotation with respect to the long axis of the propeller. In the simulation though, thermal noise is not included, so to reproduce and to study branching, simulations are run with different initial configurations of the propellers, i.e., different orientations of the propeller in the lab system and respect to the rotating magnetic field. To determine branches systematically, we run simulation that walk along the branches as in a hysteresis curve: the frequency of the magnetic field increases in steps using the final configuration of one simulation as initial configuration for the next simulation, thus very likely staying on the same branch. When the highest frequency to be simulated is reached, the procedure is repeated decreasing the frequency, typically exploring the other branch.

### 2.7. Determination of step-out frequency

To determine the step-out frequency *f*_so_ in the simulations, we check if the frequency of the propeller *f*_CM_ is synchronized with the frequency of the magnetic field *f*_B_. If oscillations of the propeller frequency *f*_CM_(*t*) can be seen in time instead of the constant value given by *f*_B_, then the asynchronous regime has started. The frequency at which the change happens is the step-out frequency. See Supplementary Information for example plots.

## 3. Results for helical propellers

Inspired by the rotating bacterial flagella, helical propellers are the best-studied type of micropropellers, both from theoretical and computational points of view (Ghosh et al., 2012, 2013; Keaveny et al., 2013; Man and Lauga, 2013; Morozov and Leshansky, 2014; Morozov et al., 2017), and with respect to experimental observations (Ghosh and Fischer, 2009; Ghosh et al., 2012; Tottori et al., 2012). Thus, we use a helical propeller as a model system to test our approach.

### 3.1. Magnetization along the short axis

**Figure 1:**
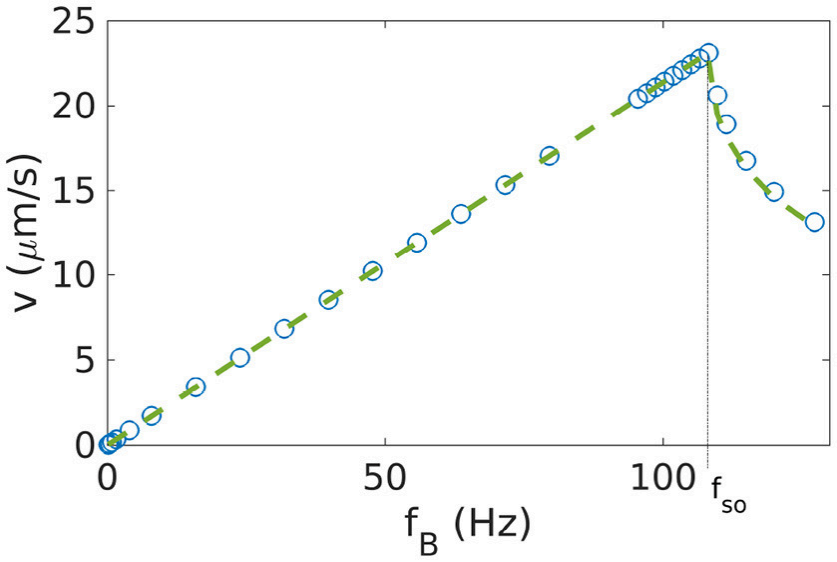
Velocity-frequency curve for a helix with $\theta_{\text{mag}}=0^{{}^{\circ}}$. The simulations (data points) are well described by Equation 10 (dashed line). To obtain the parameters for that expression, we determined *f*_so_ as described in section 2.7 and *c*_v_ from a linear fit to the behavior for *f*_*B*_ < *f*_so_.

We start with the simplest case where the magnetic moment of the helical propeller is perpendicular to the long axis of the helix. The angle between the short axis of the helix and the magnetic moment is thus θ_mag_ = 0. We confirm previous results showing that the behavior of such a helix presents two distinct regimes (Debora et al., 2013; Ghosh et al., 2013; Vach et al., 2013; Fu et al., 2015; Morozov et al., 2017), depending on the frequency with which the magnetic field rotates. For small frequencies, the helical propeller rotates in a synchronized fashion with the external field (see video 1 of Supplementary Information). The propeller velocity is proportional to the frequency of the rotating field *v*_CM_ = *c*_v_*f*_B_, where *c*_v_ is the linear coupling. The helix does not necessarily rotate exactly around its long axis, but may present a wobbling angle θ_wobb_ between the long axis and the axis of rotation of the magnetic field (Ghosh et al., 2012; Man and Lauga, 2013). Above a critical frequency, the so-called step-out frequency *f*_so_, the synchronization is lost (Ghosh et al., 2013) and the propeller cannot keep up with the external field (see videos 2, 3 of Supplementary Information). Thus, the velocity drops above the step-out frequency (at which the maximal velocity *v*_m_ = *v*_CM_(*f*_so_) is achieved). Here the wobbling angle is not constant, but oscillates. For more general propeller shapes and for helices with other orientations of the magnetic moment, the synchronous regime is divided further, as will be discussed below (see sections 3.3 and 4). Figure 1 shows the results for θ_mag_ = 0 from our simulations, which indeed exhibit the synchronous and asynchronous regimes. Thus, the behavior shown in Figure 1 can be characterized by three parameters: the maximal velocity *v*_m_, the step-out frequency *f*_so_, and the linear coupling *c*_v_ ≃ *v*_m_/*f*_so_. When two of these are read out from the simulation data, the velocity-frequency relation is fully determined and given by (Vach et al., 2013)

(10) $$\begin{array}{l} v_{\text{CM}}=\left\{ \begin{array}{cc} c_{\text{v}}f_{\text{B}}\; & \text{if}f_{\text{CM}}\leq f_{\text{so}}\\ c_{\text{v}}\left(f_{\text{B}}-\sqrt{f_{\text{B}}^{2}-f_{\text{so}}^{2}}\right)\; & \text{if}f_{\text{CM}}\geq f_{\text{so}}. \end{array}\right. \end{array}$$

For the helix shown in Figure 1, the simulation data are in excellent agreement with this functional form. The coupling coefficient *c*_v_ assumes a particularly simple form for a one-dimensional system where the axis of rotation is not changing (such as for an helix with the magnetic moment along the short axis). The coupling coefficient becomes $c_{\text{v}}=2\pi \mu^{\text{tr}}/\mu^{\text{rr}}$ (Vach, 2015), where the mobility matrix components μ^tr^ and μ^rr^ are the rotation-translation coupling and the rotation rotation coupling, respectively. For more complex cases, it is not easy to obtain a simple form for the coupling coefficient.

### 3.2. Influence of the simulation parameters

**Figure 2:**
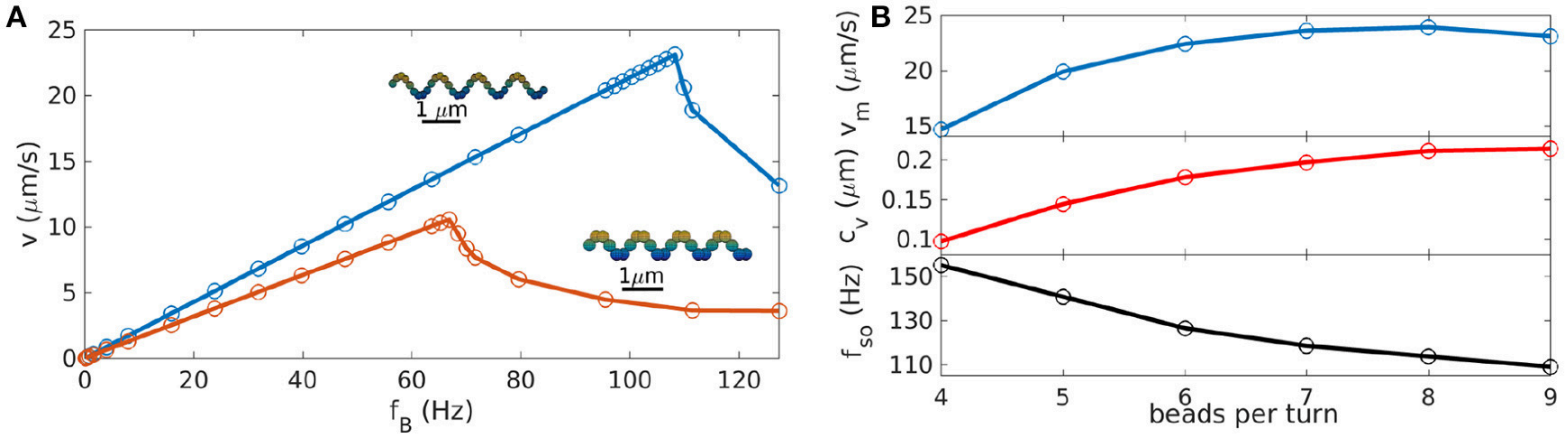
Impact of simulation parameters on the results for helices (with θ_mag_ = 0°). **(A)** Velocity-frequency curve for helices represented with beads of different sizes, *a* = 0.1μm with 9 beads per turn (blue) and , *a*′ = 1.5*a* with 6 beads per turn (red). Additional frequency-velocity curves for other bead-sizes and plots of the behavior of coupling coefficient, maximum velocity and step-out frequency can be found in the Supplementary Information. **(B)** Parameters of the velocity-frequency curve as functions of the distance between beads: step-out frequency *f*_so_, maximal velocity at the step-out *v*_m_ and linear coupling coefficient *c*_v_. The case of 9 beads per turn corresponds to beads touching each other (*a* = 0.1 μm).

Next, the influence of the simulation parameters on the results were tested, in particular the parameters related to the discretization of the propeller. Therefore, we systematically vary the bead size and the number of beads per turn of the helix (and thus the distance between beads). Changing the bead size (while keeping the gap size between beads the same) has two effects: on the one hand, it changes the discretization of the helix, which ideally should not affect the simulation results, as different discretizations describe the same geometry. On the other hand, it also changes the thickness of the helix and thereby the area of the surface in contact with the fluid, increasing its friction from where a true physical effect is expected. To quantify this difference, we performed simulations for two helices discretized by different bead sizes, but with all other geometric parameters the same. This results in the two frequency-velocity relations that we show in Figure 2A. Here we have chosen a helix with *n* = 4, *r* = 2.5μm, *p* = 4*r* and a gap size of 0 between beads (our standard parameters). The two bead sizes are *a* = 0.1 μm and *a*′ = 1.5*a*. To maintain the gap sizes of zero (i.e., beads touching each other), we need to adjust the number of beads per turn (9 in the first case, 6 in the second, see the insets in Figure 2A). The velocity-frequency curve is the same, only with scaled down values for bigger beads for which both the step-out frequency and the maximal velocity are reduced compared to the case with smaller beads (Figure 2A).

This can be explained by the higher friction arising from the increased surface area and should be considered as a result of the change in helix geometry rather than a discretization effect. Thus, to match experimental data, these effects should be taken into account. For example, beads with a diameter corresponding to the thickness of the helix can be used. Alternatively, an approximation of the real thickness of the helix can be implemented with multiple parallel chains of smaller beads instead of the one chain of large beads.

The choice of the number of beads per turn (for constant bead size) does not change the geometry of the helix, but only how the helix is represented in the discretized model: fewer beads should result in a poorer representation of the continuous surface of the helical propeller, while for more beads and thus smaller gaps, the Rotne-Prager approximation becomes invalid. To test the influence of the number of beads per turn, we approximate the same helix with different numbers of beads that are equally distributed along the curve (*n*_pt_ = 4 − 9 beads per turn of the helix). For all different discretizations, we determined frequency-velocity curves and extracted the three characteristic parameters *f*_so_, *v*_m_, and *c*_v_. These are plotted in Figure 2B as functions of the number of beads per turn. All three parameters show a dependence on the discretization, which however, saturates for large *n*_pt_. In particular, a discretization with beads touching each other (*n*_pt_ = 9) gives essentially the same results as the case *n*_pt_ = 6, where the gap is big enough to use the Rotne-Prager approximation. For large gaps however, the velocity is noticeably reduced, indicating that the continuous structure of the helix is poorly represented. Thus, a sufficient density of beads is needed for a good representation of the continuous geometry, but the precise choice of the discretization is unimportant if the beads are sufficiently dense. For the further study of helices we therefore used beads touching each other.

### 3.3. Impact of the orientation of the magnetic moment: tumbling and velocity reversals

**Figure 3:**
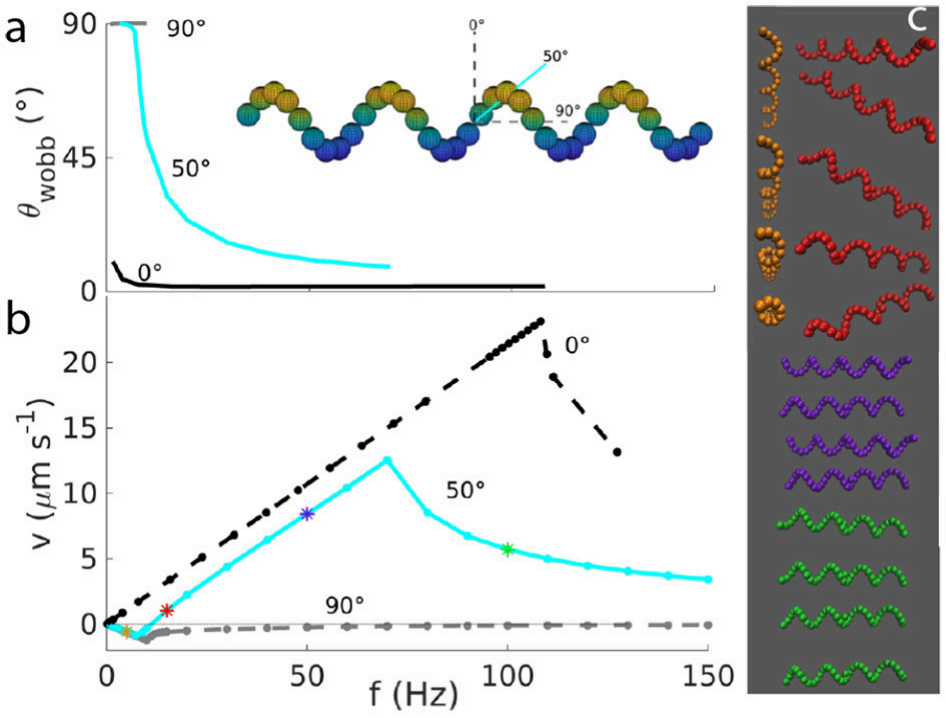
**(a)** Wobbling angle and **(b)** velocity as functions of the frequency for helices with different orientations of the magnetic moment ($\theta_{\text{mag}}=0,50,90^{{}^{\circ}}$, as depicted in the inset). The wobbling angle is defined only up to the step-out frequency. **(c)** Snapshots of the helix motion for frequencies of 5 Hz (yellow), 15 Hz (red), 50 Hz (violet), 100 Hz (green) illustrate tumbling at 5 Hz, wobbling at 15 and 50 Hz, and asynchronous rotation at 100 Hz.

Next, we change how the helix is magnetized, varying the magnetization angle θ_mag_ with respect to the short axis of the helix (see inset in Figure 3). A magnetization perpendicular to the long helix axis thus corresponds to $\theta_{\text{mag}}=0^{{}^{\circ}}$. For angles $\theta_{\text{mag}}>0^{{}^{\circ}}$, in addition to the linear regime and the asynchronous regime described by Equation 10, a third regime appears for very small frequencies. In this regime, the helix rotates along the short axis of the body, with a wobbling angle of 90°. This regime was first demonstrated experimentally (Ghosh et al., 2012), but in that case the velocity of the propeller was zero. As we can see by our simulations, and as previously shown by Morozov et al. (2017), a non-zero negative velocity is possible in this regime. To define tumbling, we take into consideration the wobbling angle θ_wobb_, that is the angle between the main axis of the helix and the axis of rotation of the helix. This angle is 90° for tumbling and it would be 0° for an helix aligned with the axis of the magnetic field.

There are two limiting cases in this three-regimes description. The first is $\theta_{\text{mag}}=0^{{}^{\circ}}$ (and equivalently 180°), for which we do not have the tumbling regime, as we can see from its wobbling angle always close to zero (black lines of Figure 3A). The second case is $\theta_{\text{mag}}=90^{{}^{\circ}}$ (and similarly for 270°), for which we obtain a very similar velocity-frequency curve as for $\theta_{\text{mag}}=0^{{}^{\circ}}$, but with all negative velocities (and smaller absolute values). In this case, the helix tumbles for all frequencies below the step-out frequency. For these frequencies, the wobbling angle approximately 90° (gray lines of Figure 3).

For all the other angles, different from 0, 90, 180, 270°, we observe both negative and positive velocities. A typical velocity-frequency curve is shown for $\theta_{\text{mag}}=50^{{}^{\circ}}$ in Figure 3 (light blue lines), with the corresponding wobbling angle. Here the three regimes can be identified. The first regime is found at very low frequencies, where the velocity is negative and decreases linearly up to a minimal velocity. In this regime, the helix tumbles, with θ_wobb_ close to 90°. This means that the propeller rotates along its short axis, as it can be seen by the yellow snapshots at 5 Hz in Figure 3. After this minimum, the velocity increases again and becomes positive for higher frequencies, where the velocity-frequency relation resembles the one for the helix with magnetization perpendicular to the long helix axis and shows the second regime, which is semi-linear and synchronous. In this second regime, the wobbling angle decreases (compare the red snapshot at 15 Hz and the violet one at 50 Hz in Figure 3). After the step-out frequency, the third (asynchronous) regime begins, where the rotation of the propeller cannot keep up with the applied frequency corresponding to a drop in the velocity. The wobbling angle oscillates (see Supplementary Information for a plot of the angle in time), due to the oscillation of the propeller-rotation-axis (green snapshot at 100 Hz in Figure 3). The oscillation of the wobbling angle results in a corresponding modulation of the rotation frequency of the propeller (see Supplementary Information).

**Figure 4:**
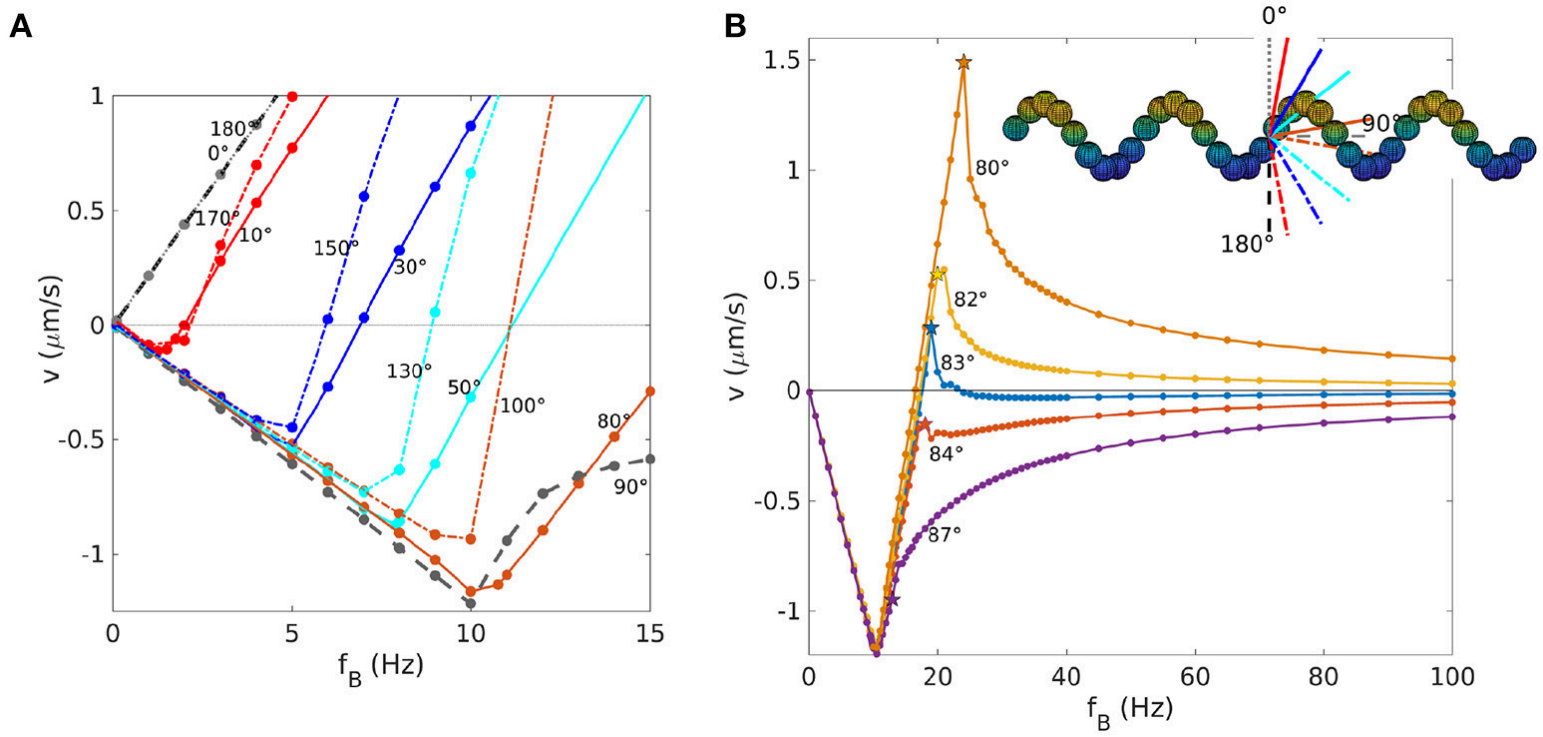
Velocity vs. frequency for helices with different θ_mag_. **(A)** Behavior for small frequencies with positive and negative velocities. Curves with the same color have mirrored orientations with respect to the main axis of the helix. **(B)** Dependence of the behavior in the asynchronous regime on the orientation of the magnetization. The limit *v* = 0 for large frequencies can be reached from above or below. Note the velocity reversal for the case of 83°, with a positive step-out velocity (indicated by a star) and negative velocities at large frequencies.

In Figure 4A, we show the behavior for very low frequencies in more detail and for more values of the angle θ_mag_. The depth of the negative peak of velocities depends on the orientation of θ_mag_ (Figure 4A). The minima of the velocity for $0^{{}^{\circ}}<\theta_{\text{mag}}<90^{{}^{\circ}}$ appear to lie on a straight line *v*_min_ = *c*_v, −_*f*_min_ with *c*_v, −_ ≃ −0.12μm. With this, we can derive a dimensionless velocity *U*_−_ for the propulsion in negative direction. The dimensionless velocity is defined as $U=1,000\times \frac{v}{Lf}$, which here becomes *U*_−_ = 1, 000*c*_v, −_/*L*. With a characteristic length of the propeller of *L* ≃ 4μm, we obtain *U*_−_ ≃ −30. This dimensionless velocity is independent of the polar angle θ_mag_ = 0 (but changes of the azimuthal angle could lead to changes Morozov et al., 2017). The dimensionless velocity *U*_+_ achieved in positive direction is approximately twice *U*_−_, thus this negative velocity is significant. Interestingly, for the case $\theta_{\text{mag}}=90^{{}^{\circ}}$ case, the velocity-frequency curve deviates from the behavior at intermediate angle, thus this case was not included in the fit to determine *c*_v, −_. For angles between 100 and 180°, a similar linear behavior is seen, but with a different slope. We highlight that the behavior between 0 and 90° differs from the behavior between 90 and 180° (Figure 4A) (Morozov et al., 2017), while the behavior between 0 and 180° is replicated between 180 and 360°. This shows that the key parameter for propulsion is the axis of the magnetic moment and not the orientation along that axis. Thus, 80° corresponds to 100°, for example.

### 3.4. Impact of the orientation of the magnetic moment: the asynchronous regime

**Figure 5:**
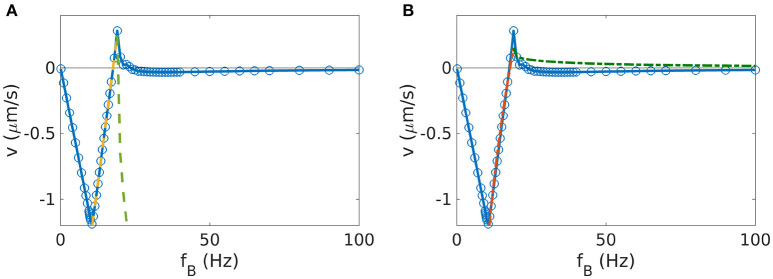
Fit of the step out behavior for a helix with $\theta_{\text{mag}}=83^{{}^{\circ}}$. The simulated data are shown in blue. **(A)** Fit function from Equation 10. The yellow line represents the linear fit below the step-out frequency *f*_so_, which was determined as described in section 2.7. This linear fit gives the constant *c*_v_, which was used to plot the green curve after step-out following equation 10. **(B)** Fit function proposed in ref. (Morozov and Leshansky, 2014); red line for the fit below the step-out frequency, green line for the corresponding behavior after step-out.

We have seen how the orientation of the magnetic moment influences the behavior of the propeller at frequencies below the step-out frequency. We now concentrate on the influence on the asynchronous regime (Figure 4B). We have seen in section 3.1 how the asynchronous behavior of the velocity curve is fully described by Equation 10 for θ_mag_ = 0 (Figure 1). However, for angles of magnetization θ_mag_ close to 90°, this formula does not describe the data well (an example for $\theta_{\text{mag}}=83^{{}^{\circ}}$ in Figure 5A). We also tried to fit the data with another expression (Morozov and Leshansky, 2014), which is valid in the asynchronous regime for frequencies near the step-out frequency,

(11) $$\begin{array}{l} v_{\text{CM}}=\left\{ \begin{array}{c} c_{\text{v}}f_{\text{B}}\left(1-f_{\text{c}}^{2}/f_{\text{B}}^{2}\right)\text{ if }f_{\text{B}}\leq f_{\text{so}}\;\\ c_{\text{v}}f_{\text{B}}\left(1-f_{\text{c}}^{2}/f_{\text{B}}^{2}\right)\left(1-\sqrt{f_{\text{B}}^{2}-f_{\text{so}}^{2}}/\sqrt{f_{\text{B}}^{2}-f_{\text{c}}^{2}}\right)\text{ if }f_{\text{B}}\geq f_{\text{so}}.\; \end{array}\right. \end{array}$$

The parameters *c*_v_ and *f*_c_ can be determined from the behavior for *f*_B_ ≤ *f*_so_. This expression also does not describe the simulation data (Figure 5B). In particular, we notice that the velocity approaches 0 in the high frequency limit in all cases, but depending on the value of θ_mag_, it can approach it from above (for angles smaller then 83°) or from below (angles ≥83°), as shown in Figure 4B. Remarkably, for the case $\theta_{\text{mag}}=83^{{}^{\circ}}$, we observe a positive step-out velocity, but the direction of propulsion is reversed above the step-out frequency and the limit of zero velocity is approached from below. No such reversal is seen for angles of 82° or 84 degree, for which the step-out velocity and the velocity for large frequencies have the same sign (positive for 82°, negative for 84°, see Figure 4B). Thus, this behavior represents a transition that is not well described by existing theories.

## 4. Results for random-shape propellers: velocity reversals and branching

The method proposed here allows us, in principle, to simulate any shape. An example of a shape that was assembled in a random fashion is shown in section 2 of the Supplementary Information. We therefore apply our method to the randomly shaped propellers that have recently been synthesized and studied by Vach et al. (2013, 2015) and Bachmann et al. (2018). These propellers are rigid random aggregates of iron oxide nanoparticles with an intrinsic magnetic moment. For some of these propellers the swimming behavior is comparable to the one of helices with $\theta_{\text{mag}}=0^{{}^{\circ}}$ (Vach et al., 2013), although more complex behaviors are generally observed, such as branching (two different solutions at the same applied frequency) and velocity reversals. We aim here at reproducing the behavior observed in the lab, and at to study the importance of the magnetic moment orientation, a key parameter needed to predict the behavior of specifically designed propellers also for non-helical geometries.

**Figure 6:**
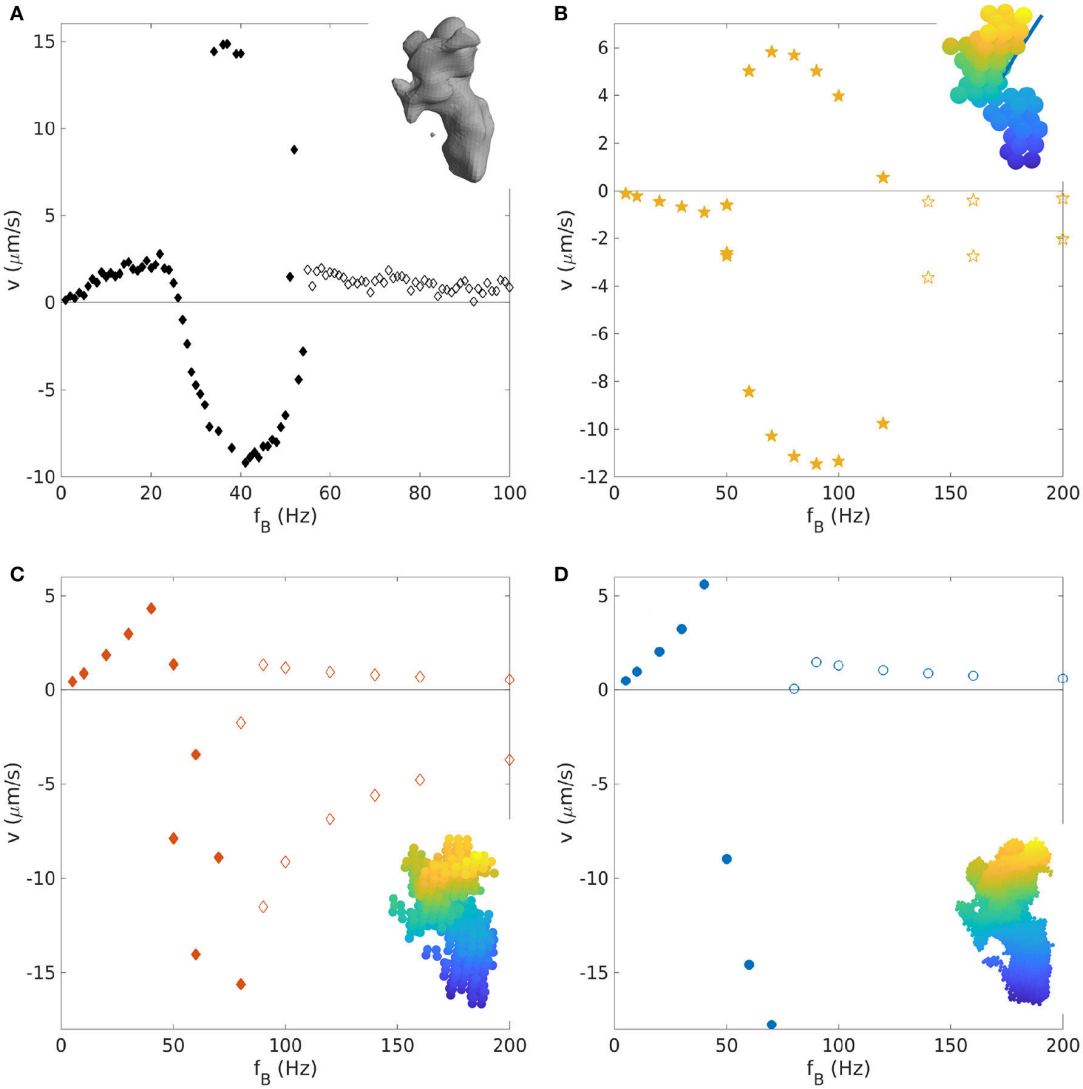
Velocity-frequency curve for a random-shaped propeller: **(A)** Experimental data (Bachmann et al., 2018); **(B–D)** Simulated velocity-frequency curve for different bead representations (shown in the insets): **(B)** 55 beads with radius *a* = 0.177μm; **(C)** 518 beads with radius *a* = 0.0903μm; and **(D)** 4276 beads with radius *a* = 0.04425μm. The magnetic moment direction is indicated as a blue line in the inset of **(B)**, and was calculated as reported in section 2.5. Filled symbols correspond to points below the step-out frequency, empty symbols to data beyond step-out.

As an example, we study a propeller geometry reported by Bachmann et al. (2018). The propeller is elongated and has an irregular surface, a geometry very different from a helix. A reconstruction of this geometry is shown as the inset in Figure 6A. For this propeller, the velocity was measured for a wide range of frequencies extending to far above the step-out frequency (Bachmann et al., 2018), shown as the black dots in Figure 6A. The absolute value and direction of the magnetic moment were calculated with the procedure described in section 2.5.

To test the influence of the discretization, we generate bead representations with different numbers and sizes of beads, from a very coarse grained representation with 55 beads to ten times more (518 beads), and finally to ~100 times more, equivalent to one bead each voxel (4,276 beads). To that end, voxels in the three-dimensional reconstruction of the propeller shape are coarse-grained as described in section 2.4. The resulting bead representations are shown as insets in the Figures 6B–D, which plot the velocity-frequency curves obtained from the corresponding simulation. None of the three representations reproduces the experimental data well, likely because the determined direction of magnetization is not precise (see below). Moreover, the results for the three representations also differ from each other. In particular, the one with 55 beads gives qualitatively different results from the other two with opposite direction of motion at low frequencies. The other two representations give similar results, thus indicating that the representation with 55 beads is too coarse-grained and that more detailed representations (≥ 500 beads) are needed to represent key features of the propeller shape. Even though the computational results in Figure 6 do not agree with the experimental data, they both show an interesting conceptual result: Branching is seen here in the synchronous and the asynchronous regime. Previous work has demonstrated bistability nearby the step-out frequency in the asynchronous regime for helices (Ghosh et al., 2012), but branching far above from the step-out was not observed.

**Figure 7:**
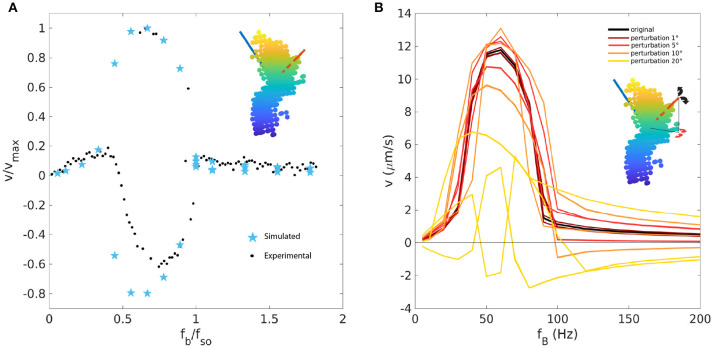
**(A)** Rescaled velocity-frequency curve for the 518 beads-approximation (light blue stars) with magnetic moment direction indicated by the red vector (best matching vector with experimental data), compared to rescaled experimental data (black dots). The direction of the magnetic moment used in the previous Figure 6 is in blue. **(B)** The direction of the magnetic moment (red vector) has been perturbed by small angles in the direction of the black and red arrows. Five random perturbation are shown as five distinct curves for each angle of perturbation.

To determine the direction of the magnetic moment, which is unknown for the experimental propeller and which could impact the propeller behavior, we run simulations for 100 random orientations of the magnetic moment using the ~500 beads-representation. The shape of the curve varies considerably within this dataset (see Supplementary Information). The 100 velocity-frequencies curves were inspected manually (see Supplementary Information) and the ones showing the basic qualitative characteristics of the experimental curves (initial positive linear behavior, positive or negative branch and positive approach to 0 after the step-out) were selected. These selected curves were then rescaled with *f*_*B*_/*f*_*so*_ and *v*/*v*_max_ and compared to the rescaled experimental data. The rescaling is necessary to correct for possible shifts of the velocity-frequency curve due to the approximations and to take the unknown absolute value of the magnetic moment into account. The closest match between computational and experimental curves was found for the magnetization direction shown as the red vector in Figure 7 was selected, which is considerably different from the direction calculated with the method describes in section 2.5 (blue vector). With this direction of magnetization, the experimental data are described quite well, although some discrepancies remain in the negative branch (Figure 7A).

Since the direction of the magnetization emerges as a crucial parameter needed to describe the experimental behavior, we tested how sensitive the results are to changes in this direction, by making small perturbations to it (Figure 7B). For perturbation of ±1°, the curve remains the same with only small variations. For perturbations of 5°, the curve maintains its main (qualitative) characteristics, while shifts in velocity are seen. For perturbations exceeding 5°, strong deviations are seen, for example different branching behavior. Thus, we conclude that, for this geometry, the direction of the magnetization should be known with an accuracy of about 5° or better.

## 5. Discussion

The aim of this study was to develop a method for simulating magnetic micropropellers with complex shapes in order to predict their behavior, specifically their propulsion velocity and its dependence on the strength and frequency of the magnetic field actuating these propellers. Such a simulation method is desirable for optimizing the design of magnetic microswimmers for specific purposes or applications, as magnetically actuated microswimmers are envisioned to perform tasks such as drug delivery, fertilization, artery cleaning, biopsies *etc*. (Peyer et al., 2013; Magdanz and Schmidt, 2014; Sitti et al., 2015; Stanton et al., 2015; Schwarz et al., 2017). The variety of tasks and environments may require the customization of the swimmer with desired characteristics such as high speed, rapid change of direction, ease of control, etc., which are likely not all achievable with the helical propeller geometry that has dominated the early phase of propeller development. Simulations offer a way to study and optimize existing random geometries as well as to design new geometries with desired characteristics.

We first used the method to simulate the well-studied helical geometry, for which our simulations successfully recovered the known behavior. Small shifts in the velocity-frequency curve arise from the choice of the mobility matrix and approximations involved in it. This could be improved by re-designing the matrix elements. The systematic study on the influence of the simulation parameters such as bead dimension and bead separation suggests that beads touching each other can be used despite the use of the Rotne-Prager approximation, but this must be done cautiously, depending on the way a shape is represented. Changing the dimension and number of beads could lead to different results, so the discretization of the original shape must be done such as not to change the main features of the propeller geometry.

We have then used the model to explore aspects of helical propellers that have not received much attention before, in particular, the influence of the orientation of the magnetic moment, which can result in velocity reversals both below and above the step-out frequency. Velocity reversals are accompanied by a change of propeller orientation relative to its direction of motion. Velocity reversals for helices at small frequencies were not seen experimentally yet, but have been seen in recent theoretical work and simulations (Morozov et al., 2017). Velocity reversal at high frequencies, above the step-out frequency have not been reported before (and the behavior above the step-out frequency is in general not well described by existing theories Vach et al., 2013; Morozov and Leshansky, 2014, emphasizing how this regime is still poorly studied). In general velocity reversals are of interest for applications, because they enable diverse modes of actuation and provide additional mechanism to steer propellers. Such reversals are not only characteristic for helices, but are also seen for random-shaped propellers, whose geometry can enhance or reduce this property. The results for helices underline the importance of the direction of the magnetic moment, which can change the dynamics of the propeller considerably.

While the results we obtain for helices are rather robust with respect to the representation of the propeller shape, this is less so for the random-aggregate propeller we studied next. These simulations show that it is crucial to reconstruct the propeller shape accurately as well as to know the orientation of the magnetic moment, extending previous results (Ghosh et al., 2012; Morozov et al., 2017). Both of these factors could completely change the dynamics of the system, such as step-out frequencies, magnitude of velocities, branching and switching between positive and negative velocities. The main challenges here are the discretization of the propeller and the determination of the magnetic moment for existing geometries. If measuring the magnetic moment is not possible, simulations provide an alternative by simulating many orientations to find the best fit to experimental velocity-frequency relations. By comparison, a previously proposed method to determine magnetic moment via a cylindrical approximation (Ghosh et al., 2013) gave us poor result for our bead-approximated geometries inferred for an experimentally realized propeller. As for the bead discretization, we propose a method to match the shape obtained by a 3D-reconstruction from experimental images. This method is highly reproducible and allows also the choice of the number and dimension of beads. We show that very coarse grained discretization, with fewer bigger beads, could change the behavior of the propeller, while there is saturation for higher number of beads (500 and 4700 beads behave the same way).

All these results highlight the importance of accurately determining the orientation of the magnetic moment, on which most of the dynamics depend. Not only is this needed to understand and predict the behavior of existing geometries, but it can also be used to design new propellers with the required characteristics: not only shape must be taken into account, but magnetization too. However, designing the magnetization direction constitutes another challenge, as a design that works well in simulations may be difficult or even impossible to synthesize in the lab. The most promising technique to that end is 3D printing, with which in principle any shape can be implemented. However, the magnetization represents another problem. Existing techniques such as different coating approaches (sputtering etc. Fischer and Ghosh, 2011) or the use of magnetized beads as component of the microswimmer (see for helix Fischer and Ghosh, 2011) can give a rough estimate of the direction of magnetization, but could not achieve the required precision of ~5° to have fully predictable behavior. If customized micropropellers are envisioned in the future of bio-medicine, greater attention must be focused on the magnetization process, in addition to the shape characterization.

## Author contributions

AC, DF, and SK designed research, AC wrote the code and performed simulations. All authors analyzed data and wrote the manuscript.

### Conflict of interest statement

The authors declare that the research was conducted in the absence of any commercial or financial relationships that could be construed as a potential conflict of interest.

## References

[B1] Abbott J. J. Peyer K. E. Lagomarsino M. C. Zhang L. Dong L. Kaliakatsos I. K. (2009). How should microrobots swim? Int. J. Rob. Res. 28, 1434–1447. 10.1177/0278364909341658

[B2] Bachmann F. Bente K. Codutti A. Faivre D. [Preprint] (2018). Using shape diversity on the way to new structure-function designs for magnetic micropropellers. *arXiv:1809.01023*. Available online at: https://arxiv.org/abs/1809.01023

[B3] Brady J. F. Bossis G. (1988). Stokesian dynamics. Ann. Rev. Fluid Mechan. 20, 111–157. 10.1146/annurev.fl.20.010188.000551

[B4] Carrasco B. de la Torre J. G. (1999). Hydrodynamic properties of rigid particles: comparison of different modeling and computational procedures. Biophys. J. 76, 3044–3057. 10.1016/S0006-3495(99)77457-6 10354430 PMC1300274

[B5] Cheang U. K. Meshkati F. Kim D. Kim M. J. Fu H. C. (2014). Minimal geometric requirements for micropropulsion via magnetic rotation. Phys. Rev. E 90:033007. 10.1103/PhysRevE.90.033007 25314529

[B6] Debora S. Marcel P. John G. G Björn M. Andrew G. M Peer F. (2013). Chiral colloidal molecules and observation of the propeller effect. J. Am. Chem. Soc. 135, 12353–12359. 10.1021/ja405705x 23883328 PMC3856768

[B7] Dhont J. K. (1996). An Introduction to Dynamics of Colloids. Amsterdam: Elsevier.

[B8] Elgeti J. Winkler R. G. Gompper G. (2015). Physics of microswimmers single particle motion and collective behavior: a review. Rep. Prog. Phys. 78:056601. 10.1088/0034-4885/78/5/056601 25919479

[B9] Filippov A. (2000). Drag and torque on clusters of n arbitrary spheres at low reynolds number. J. Colloid Interface Sci. 229, 184–195. 10.1006/jcis.2000.6981 10942557

[B10] Fischer P. Ghosh A. (2011). Magnetically actuated propulsion at low reynolds numbers: towards nanoscale control. Nanoscale 3, 557–563. 10.1039/C0NR00566E 21152575

[B11] Fu H. C. Jabbarzadeh M. Meshkati F. (2015). Magnetization directions and geometries of helical microswimmers for linear velocity-frequency response. Phys. Rev. E 91:043011. 10.1103/PhysRevE.91.043011 25974584

[B12] Ghosh A. Fischer P. (2009). Controlled propulsion of artificial magnetic nanostructured propellers. Nano Lett. 9, 2243–2245. 10.1021/nl900186w 19413293

[B13] Ghosh A. Mandal P. Karmakar S. Ghosh A. (2013). Analytical theory and stability analysis of an elongated nanoscale object under external torque. Phys. Chem. Chem. Phys. 15, 10817–10823. 10.1039/c3cp50701g 23694848

[B14] Ghosh A. Paria D. Singh H. J. Venugopalan P. L. Ghosh A. (2012). Dynamical configurations and bistability of helical nanostructures under external torque. Phys. Rev. E 86:031401. 10.1103/PhysRevE.86.031401 23030914

[B15] Helgesen G. (2018). Magnetic propulsion of microspheres at liquid-glass interfaces. J. Appl. Phys. 123:064902 10.1063/1.5011350

[B16] Hinsen K. (1995). Hydrolib: a library for the evaluation of hydrodynamic interactions in colloidal suspensions. Comput. Phys. Commun. 88, 327–340. 10.1016/0010-4655(95)00029-F

[B17] Janssen X. Schellekens A. van Ommering K. van IJzendoorn L. Prins M. (2009). Controlled torque on superparamagnetic beads for functional biosensors. Biosens. Bioelectron. 24, 1937–1941. 10.1016/j.bios.2008.09.024 19022651

[B18] Keaveny E. E. Walker S. W. Shelley M. J. (2013). Optimization of chiral structures for microscale propulsion. Nano Lett. 13, 531–537. 10.1021/nl3040477 23317170

[B19] Lauga E. (2016). Bacterial hydrodynamics. Ann. Rev. Fluid Mech. 48, 105–130. 10.1146/annurev-fluid-122414-034606

[B20] Magdanz V. Schmidt O. G. (2014). Spermbots: potential impact for drug delivery and assisted reproductive technologies. Exp. Opin. Drug Deliv. 11, 1125–1129. 10.1517/17425247.2014.924502 24882224

[B21] Man Y. Lauga E. (2013). The wobbling-to-swimming transition of rotated helices. Phys. Fluids 25, 071904 10.1063/1.4812637

[B22] Manghi M. Schlagberger X. Netz R. R. (2006). Propulsion with a rotating elastic nanorod. Phys. Rev. Lett. 96:068101. 10.1103/PhysRevLett.96.068101 16606051

[B23] Martinez-Pedrero F. Ortiz-Ambriz A. Pagonabarraga I. Tierno P. (2015). Colloidal microworms propelling via a cooperative hydrodynamic conveyor belt. Phys. Rev. Lett. 115:138301. 10.1103/PhysRevLett.115.138301 26451584

[B24] Mirzae Y. Dubrovski O. Kenneth O. Morozov K. I. Leshansky A. M. (2018). Geometric constraints and optimization in externally driven propulsion. Sci. Rob. 3:eaas8713 10.1126/scirobotics.aas8713 33141739

[B25] Morozov K. I. Leshansky A. M. (2014). The chiral magnetic nanomotors. Nanoscale 6, 1580–1588. 10.1039/C3NR04853E 24336860

[B26] Morozov K. I. Mirzae Y. Kenneth O. Leshansky A. M. (2017). Dynamics of arbitrary shaped propellers driven by a rotating magnetic field. Phys. Rev. Fluids 2:044202 10.1103/PhysRevFluids.2.044202

[B27] Ortega A. de la Torre J. G. (2003). Hydrodynamic properties of rodlike and disklike particles in dilute solution. J. Chem. Phys. 119, 9914–9919. 10.1063/1.1615967

[B28] Ouajdi F. Mahmood M. Samira T. Dominic d. L. Yong Z. X. Dumitru L. (2016). Magneto-aerotactic bacteria deliver drug-containing nanoliposomes to tumour hypoxic regions. Nat. Nanotechnol. 11:941 10.1038/nnano.2016.137 27525475 PMC6094936

[B29] Peyer K. E. Zhang L. Nelson B. J. (2013). Bio-inspired magnetic swimming microrobots for biomedical applications. Nanoscale 5, 1259–1272. 10.1039/C2NR32554C 23165991

[B30] Press W. H. Teukolsky S. A. Vetterling W. T. Flannery B. P. (1992). Numerical Recipes in Fortran 2nd Edn., The Art of Scientific Computing. New York, NY: Cambridge University Press.

[B31] Reichert M. (2006). Hydrodynamic Interactions in Colloidal and Biological Systems. Dissertation, Konstanz University.

[B32] Rotne J. Prager S. (1969). Variational treatment of hydrodynamic interaction in polymers. J. Chem. Phys. 50, 4831–4837. 10.1063/1.1670977

[B33] Sachs J. Morozov K. I. Kenneth O. Qiu T. Segreto N. Fischer P. [Preprint] (2017). The role of symmetry in driven propulsion at low reynolds number. *arXiv:1708.01140*. Available online at: https://arxiv.org/abs/1708.01140v2

[B34] Schwarz L. Medina-Sànchez M. Schmidt O. G. (2017). Hybrid biomicromotors. Appl. Phys. Rev. 4, 031301 10.1063/1.4993441

[B35] Sitti M. Ceylan H. Hu W. Giltinan J. Turan M. Yim S.. (2015). Biomedical applications of untethered mobile milli/microrobots. Proc. IEEE 103, 205–224. 10.1109/JPROC.2014.2385105 27746484 PMC5063027

[B36] Stanton M. M. Park B.-W. Vilela D. Bente K. Faivre D. Sitti M.. (2017). Magnetotactic bacteria powered biohybrids target *E. coli* biofilms. ACS Nano 11, 9968–9978. 10.1021/acsnano.7b04128 28933815

[B37] Stanton M. M. Trichet-Paredes C. Sanchez S. (2015). Applications of three-dimensional (3d) printing for microswimmers and bio-hybrid robotics. Lab. Chip 15, 1634–1637. 10.1039/C5LC90019K 25632887

[B38] Tottori S. Nelson B. J. (2018). Controlled propulsion of two-dimensional microswimmers in a precessing magnetic field. Small 14:e1800722. 10.1002/smll.201800722 29749100

[B39] Tottori S. Zhang L. Qiu F. Krawczyk K. K. Franc-Obregón A. Nelson B. J. (2012). Magnetic helical micromachines: fabrication, controlled swimming, and cargo transport. Adv. Mater. 24, 811–816. 10.1002/adma.201103818 22213276

[B40] Vach P. (2015). Solution Synthesis and Actuation of Magnetic Nanostructures. Ph.D. thesis, Humboldt-Universität zu Berlin, Mathematisch-Naturwissenschaftliche Fakultät.

[B41] Vach P. J. Brun N. Bennet M. Bertinetti L. Widdrat M. Baumgartner J.. (2013). Selecting for function: solution synthesis of magnetic nanopropellers. Nano Lett. 13, 5373–5378. 10.1021/nl402897x 24127909 PMC3885197

[B42] Vach P. J. Fratzl P. Klumpp S. Faivre D. (2015). Fast magnetic micropropellers with random shapes. Nano Lett. 15, 7064–7070. 10.1021/acs.nanolett.5b03131 26383225 PMC4608002

[B43] Xu T. Hwang G. Andreff N. Régnier S. (2016). Influence of geometry on swimming performance of helical swimmers using doe. J. Micro-Bio Rob. 11, 57–66. 10.1007/s12213-015-0084-5

[B44] Zhang L. Abbott J. J. Dong L. Kratochvil B. E. Bell D. Nelson B. J. (2009). Artificial bacterial flagella: Fabrication and magnetic control. Appl. Phys. Lett. 94, 064107 10.1063/1.3079655

