# Supplementary material for: Bead-Based Hydrodynamic Simulations of Rigid Magnetic Micropropellers
Source: Front Robot AI. 2018 Sep 19;5:109. doi: 10.3389/frobt.2018.00109 (PMC7805997; doi:10.3389/frobt.2018.00109)
Supplement: Supplementary file 4 [file Data_Sheet_1.pdf]

Supplementary Material: Bead-based hydrodynamic simulations of rigid magnetic micropropellers

Agnese Codutti [1,2,*], Felix Bachmann [1], Damien Faivre [1] and Stefan Klumpp [2,3,*]

*Correspondence:
Stefan Klumpp
stefan.klumpp@phys.uni-goettingen.de

Agnese Codutti
agnese.codutti@mpikg.mpg.de

1 TEST OF APPROXIMATIONS IN THE MOBILITY MATRIX

To test the influence of the approximations described in the main text, we determined velocity-frequency curves for a helix with $\theta_{\rm m} = 0$ and beads touching each other in four cases with different degrees of simplifications: (a) including all terms in the mobility matrix, (b) neglecting off-diagonal rotational-rotational terms as well as rotational-translational terms, (c) neglecting off-diagonal rotational-rotational terms and rotational-translational terms and additionally approximating up to order $(a/r_{ij})^1$ only, (d) neglecting only rotational-translational terms. Figure S1 shows that the qualitative behavior, in particular the shape of the curve is the same for all cases, but we observe small shifts in both velocity and frequency. Interestingly, adding the rotational-rotational terms to (b) (thus obtaining case (d))does not change the velocity-frequency curve. The biggest shift is observed when also the rotational-translational terms are included. The quantitative differences between the approximations are relatively small. Throughout the study, we used approximation (b).

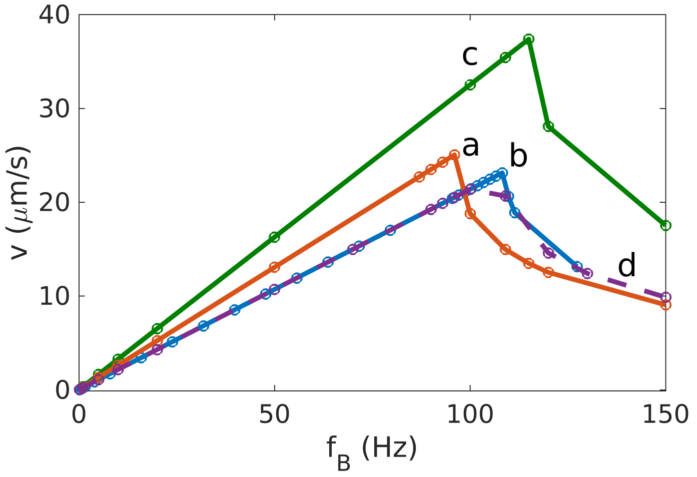

Figure S1. Influence of the approximations in the mobility matrix for a helix with $\theta_{\rm m} = 0$. The green curve corresponds to the case c), the blue curve to the case b), the red curve to the case a), and the purple dashed curve to the case d).

2 RANDOM PROPELLERS GENERATED BY A GROWTH ALGORITHM

A propeller with 60 beads of radius $a = 0.125\mu m$ was randomly generated with a growth algorithm (figure S2 inset): a first bead is placed, then a second bead is placed at a distance equal to one diameter between the centers. In the following iterations, more beads are attached to one of the previous beads, always with surface contact. Volume exclusion is applied. The final object presents a random geometry. In figure S2, we show the velocity frequency curve for such a propeller.

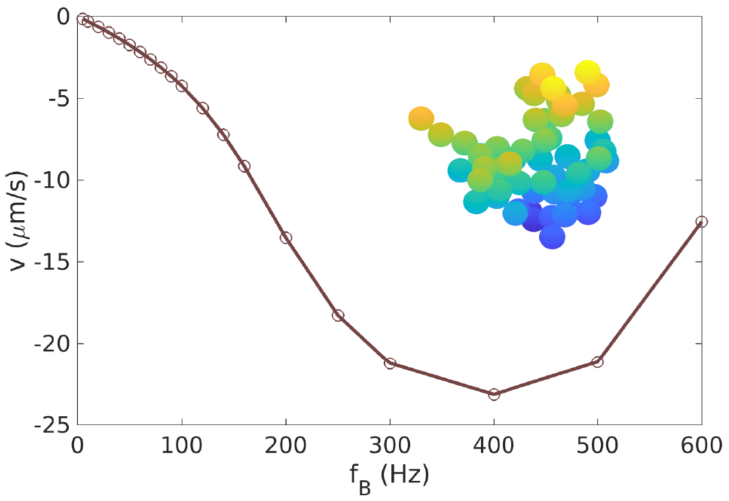

Figure S2. Velocity frequency curve for the randomly generated propeller shown in the inset (60 beads $a = 0.125\mu$m).

3 BEAD-SIZE EFFECT FOR AN HELIX

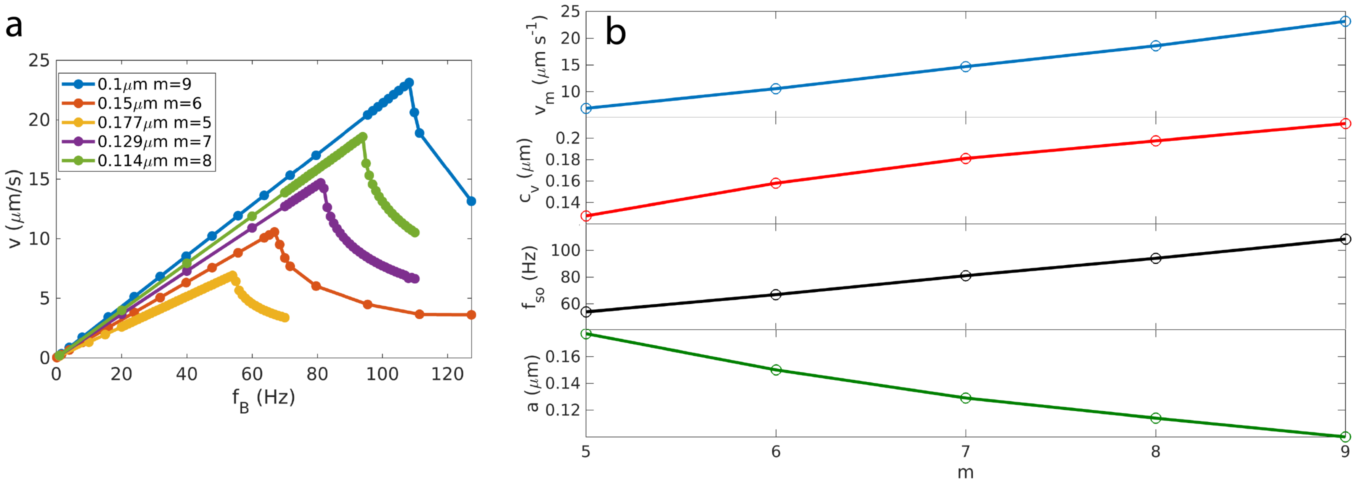

Figure S3. Effect of the bead-size for an helix with $\theta_{\mathrm{mag}} = 0°$ and touching beads: a) the velocity-frequency curves; (b) maximum velocity v_{m} (blue), coupling coefficient c_{v} (red), step-out frequency f_{so} (black) and bead-radius a (green) as function of the number of beads per turn m. Bigger beads (corresponding also to less beads per turn) shift the curve towards smaller frequencies and velocities. Changing the bead size influences not only the representation of the helix, but the helix geometry itself; bigger beads lead to a thicker helix.

4 HELIX WOBBLING ANGLE AND FREQUENCY OF THE CENTER OF MASS IN TIME

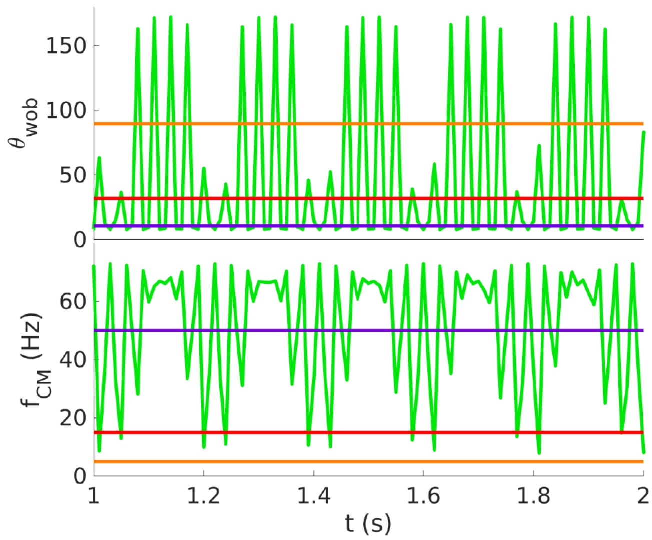

Figure S4. (a) Wobbling angle and (b) frequency of the center of mass for a helix with $\theta_{\mathrm{mag}} = 50°$ as functions of time for four different applied frequencies (5 Hz yellow, 15 Hz red, 50 Hz violet, 100 Hz green). While for frequencies below step-out both quantities are constant, they oscillated above the step-out frequency.

5 RANDOMLY SHAPED PROPELLER: RESULTS FOR 100 RANDOMLY CHOSEN DIRECTIONS OF THE MAGNETIC MOMENT

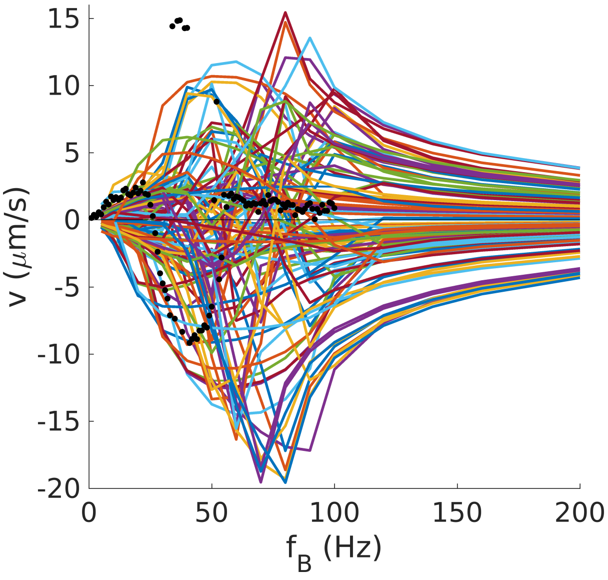

Figure S5. For the random shaped propeller described in the main text and the 518-beads discretization, propulsion is simulated with 100 randomly chosen orientations of the magnetic moment. The plot shows the curves in comparison with the un-scaled experimental data (black dots). The great impact of the magnetic moment orientation on the shape of the velocity-frequency curve can be seen.

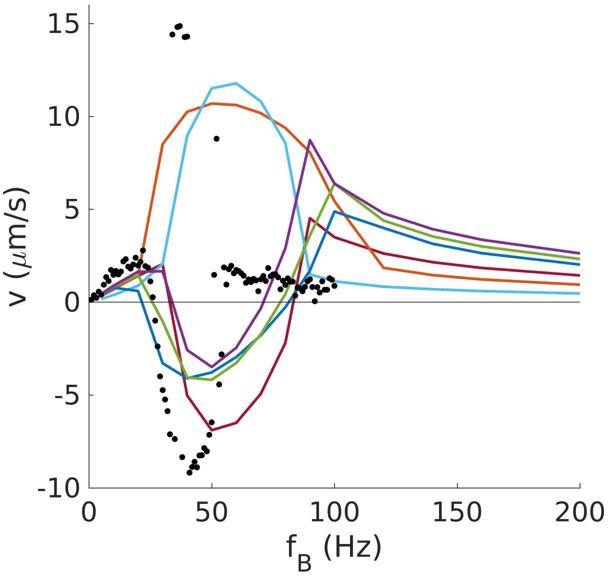

Figure S6. The magnetic moment orientations that result in the closest correspondence with the experimental data were selected based on the following criteria: presence of a positive linear regime for small frequencies, branching and a positive velocity at frequencies above step-out. The light blue curve is the best match that is used in the main text, the black dots show the experimental data.

6 VIDEOS OF HELIX BEHAVIOR BELOW AND ABOVE STEP-OUT

The three videos shows the motion of a helix with $\theta_{\mathrm{m}} = 0^{\circ}$ for different frequencies and with parameters as described in the main text. We showed only the final bead of the helix for simplicity. As can be seen in video 1, below step-out the helix rotates with the applied external frequency of 97.8Hz. Video 2 shows the asynchronous regime at 109.8Hz. Here the helix exhibits two frequencies: an effective propulsion frequency that produces the propulsion, and a twitching frequency, that makes the bead go backwards in its revolution. This effect is due to the fact that the propeller cannot keep up with the external applied frequency. Here the twitching frequency is lower then the propulsion frequency. Video 3 shows an even higher frequency of 300Hz. The behavior is the same as in Video 2, but now the twitching frequency exceeds the propulsion frequency.
